# Oligonucleotide Aptamer-Mediated Precision Therapy of Hematological Malignancies

**DOI:** 10.1016/j.omtn.2018.08.023

**Published:** 2018-09-06

**Authors:** Shuanghui Yang, Huan Li, Ling Xu, Zhenhan Deng, Wei Han, Yanting Liu, Wenqi Jiang, Youli Zu

**Affiliations:** 1 Department of Hematology, Xiangya Hospital, Central South University, Changsha 410008, Hunan, China; 2 Department of Pathology and Genomic Medicine, Houston Methodist Hospital, Houston, TX 77030, USA; 3 Department of Oncology, Xiangya Hospital, Central South University, Changsha 410008, Hunan, China; 4 Department of Hematology, First Affiliated Hospital, Jinan University, Guangzhou 510632, Guangdong, China; 5 Department of Orthopaedics, Xiangya Hospital, Central South University, Changsha 410008, Hunan, China

**Keywords:** aptamer, precision medicine, leukemia, lymphoma, multiple myeloma

## Abstract

Precision medicine has recently emerged as a promising strategy for cancer therapy because it not only specifically targets cancer cells but it also does not have adverse effects on normal cells. Oligonucleotide aptamers are a class of small molecule ligands that can specifically bind to their targets on cell surfaces with high affinity. Aptamers have great potential in precision cancer therapy due to their unique physical, chemical, and biological properties. Therefore, aptamer technology has been widely investigated for biomedical and clinical applications. This review focuses on the potential applications of aptamer technology as a new tool for precision treatment of hematological malignancies, including leukemia, lymphoma, and multiple myeloma.

## Main Text

Currently, chemotherapy is the mainstay treatment for hematological malignancies.^1^ Although chemotherapy is effective in many cases, it can cause severe side effects in patients due to a lack of specificity. Therefore, cancer cell- or oncogene-selective therapeutic approaches have recently emerged, e.g., precision medicine, as a result of the advances in cancer genotyping and phenotyping studies. Precision medicine targets specific abnormalities within the cancer cell genome, proteome, and immunome and markers involved in cancer initiation, development, and growth.^2^, ^3^ In contrast to conventional chemotherapy, precision medicine specifically targets the individual characteristics of each patient’s cancer phenotypic profile, resulting in significantly increased therapeutic efficacy and decreased non-specific toxicity. To date, precision medicine has been extensively applied in various cancers, especially hematological malignancies. The major approaches of precision medicine include the following: (1) cell-targeted chemotherapy to specifically deliver chemotherapeutic agents to the cells of interest without affecting normal tissues,^4^, ^5^ (2) gene therapy to specifically silence oncogenes,^6^, ^7^ (3) immunotherapy to harness the power of the immune system in eliminating cancers,^8^, ^9^ and (4) cell-specific biotherapies to inhibit cancer development by stimulating relevant cellular signaling pathways.^10^ Precision medicine has shown great potential and perspective in the clinical treatment of cancer as a result of the recent technological advances.

**Figure 1:**
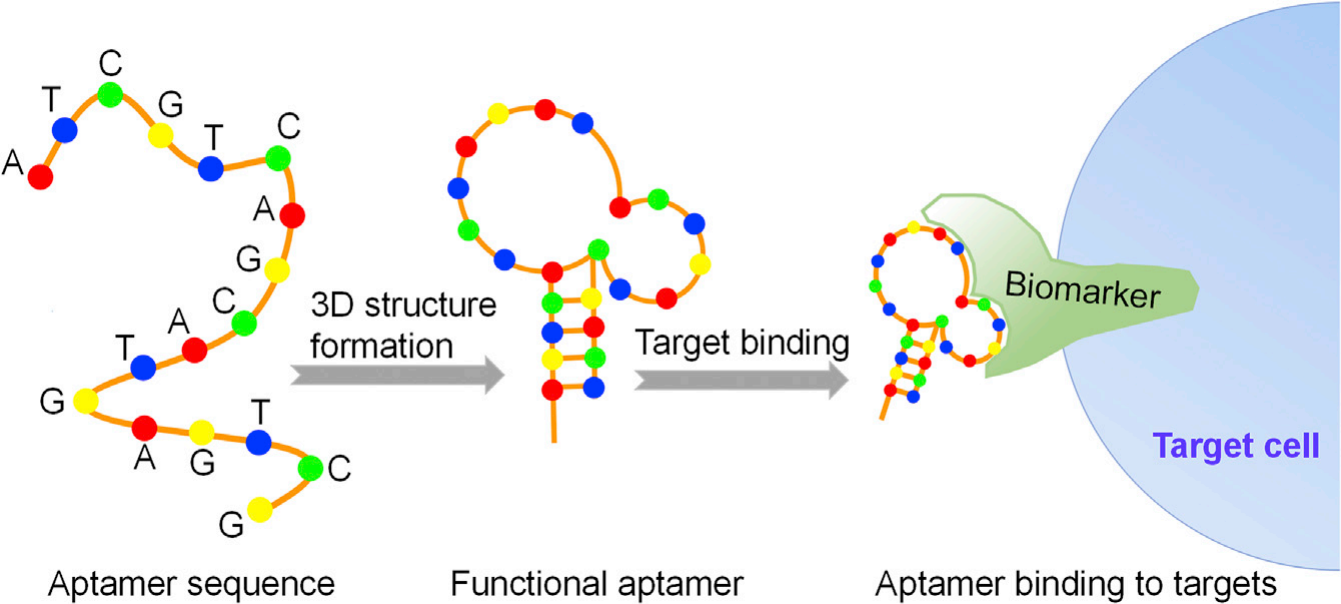
Schematic Diagram of Aptamer Function Aptamers comprising judiciously chosen oligonucleotide sequences form functional 3D structures, and they bind to their targets with high specificity and affinity.

Aptamers are short, single-stranded oligonucleotides (RNA or single-stranded DNA [ssDNA], respectively), usually 20–80 nt in length with a tridimensional folded structure. Aptamers are highly specific and bind tightly to their targets after structural recognition (Figure 1), in a manner similar to antibodies binding to their antigens.^11^ In 1990, Ellington and Szostak^12^ coined the term aptamer for this unique class of nucleic acids; the term derives from the Latin word aptus, which means fit, and the Greek word meros, which means part. To date, aptamers have been developed for a wide range of targets, including small metal ions or molecules, peptides, proteins, bacteria, viruses, whole cells, and targets within live animals.^13^ Aptamers have been widely used in the diagnosis and treatment of various diseases.^14^, ^15^, ^16^, ^17^ Oligonucleotide aptamers present the following significant advantages compared to protein antibodies:

(1) Higher tissue permeability. Aptamers have significantly smaller molecular weights (8–25 kDa) compared to antibodies (over 150 kDa);^18^ thus, they can penetrate tissue membranes and reach their target sites more efficiently *in vivo* compared to antibodies, with dissociation constants (*K*_*d*_) in the pico- to nanomolar range.^19^, ^20^ Nonetheless, the smaller molecular weights of aptamers render them susceptible to rapid excretion through renal filtration *in vivo.*^21^ During the last decade, chemical modification strategies to prevent renal clearance of aptamers have emerged, and they have significantly increased the half-life of aptamers *in vivo.*^22^ Da Pieve et al.^23^ reported that conjugation of the murine PD-1 aptamer to a high-molecular-weight polyethylene glycol (PEG) significantly limited the rate of renal filtration, and it increased the half-life of the aptamer from 1 to 24–48 hr *in vivo.* Thus, modified aptamers can exhibit high bioavailability in clinical applications, given that they readily reach their targets, and they can remain in the bloodstream for extended time periods *in vivo.*
(2) Easy to modify and low cost. Due to their simpler structures compared to antibodies, aptamers can be easily modified through chemical processes, enabling easier optimization of their clinical properties, such as resistance to nuclease degradation^24^, ^25^, ^26^ or increased half-life^27^, ^28^ *in vivo.* Aptamers can be linked with drugs, radioisotopes, RNA oligonucleotides, or even nanostructures to specifically deliver anticancer agents for targeted therapy.^29^, ^30^, ^31^ Additionally, aptamers are rapidly produced on a large scale by methods adhering to good manufacturing practices (GMPs), and they have lower production costs compared to antibodies.

**Figure 2:**
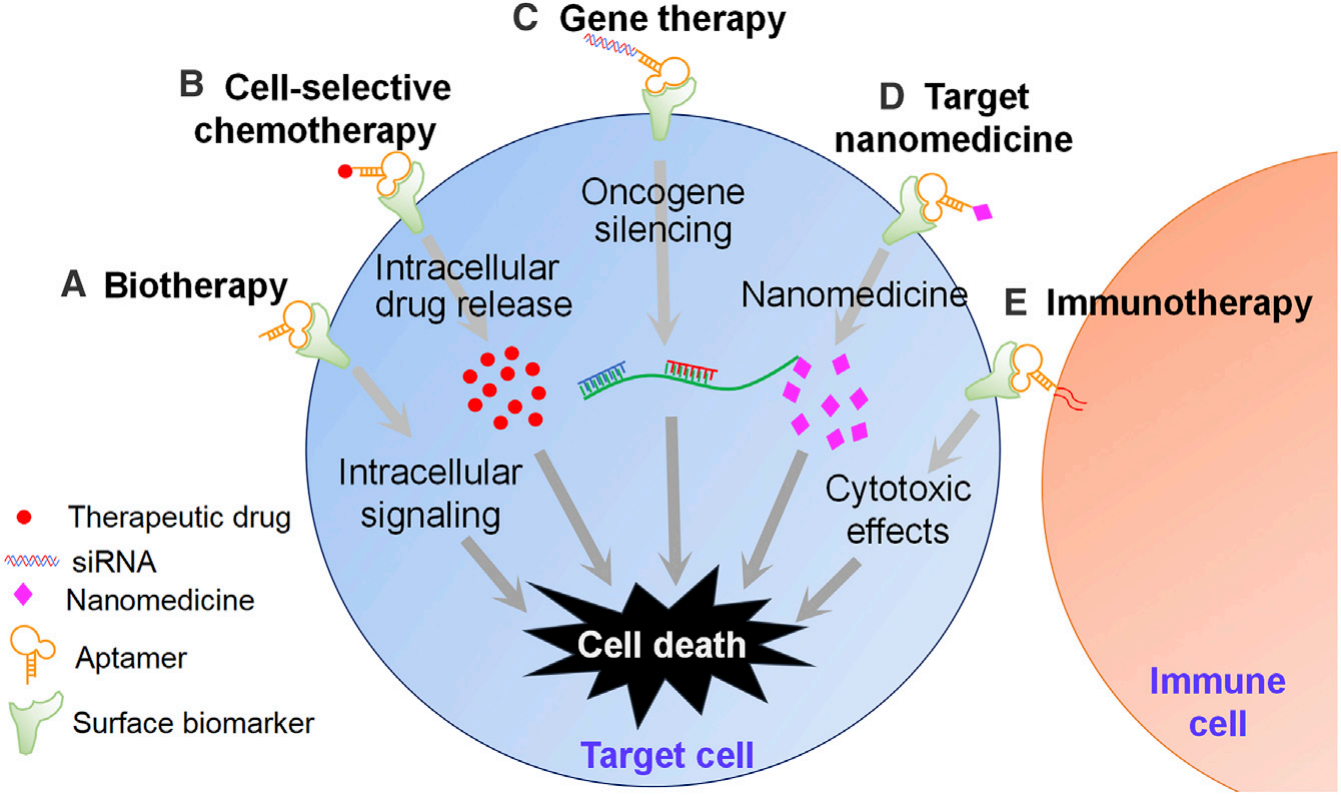
Aptamer-Mediated Precision Medicine Therapies (A) Biotherapy: aptamers bind directly to cellular surface targets, triggering cellular signaling pathways and inducing apoptosis or death of targeted cells. (B) Cell-selective chemotherapy: aptamers linked with therapeutic drugs specifically bind to cell surface targets, which results in selective intracellular drug delivery to target cells. (C) Oncogene-specific gene therapy: aptamers linked with siRNA bind to cell surface targets, thereby silencing a specific oncogene. (D) Targeted nanomedicine: aptamers linked with nanoparticles specifically bind to cellular surface targets, leading to specific intracellular delivery of cytotoxic nanomedicine to target cells. (E) Immunotherapy: immune cells equipped with aptamers specifically bind to surface biomarkers on target cells and induce cytotoxicity.

In light of the aforementioned advantages, aptamers are very promising, and they have great potential in clinical applications, rendering them a powerful tool in precision therapy of hematological malignancies. Recent advances in aptamer-based precision medicine show its superior therapeutic effects in cancer treatment as compared to conventional strategies. Each year, the increasing number of reports underscores the major advances of aptamer-based precision medicine, including biotherapy,^32^ cell-selective chemotherapy,^33^, ^34^ oncogene-specific gene therapy,^29^, ^35^ targeted nanomedicine,^36^, ^37^, ^38^ and immunotherapy (Table 1; Figure 2).^39^, ^40^

**Table 1:** Aptamers Specifically Targeting Cell Surface Biomarkers Studied for Precision Cancer Therapy

Approach	Mechanism	Effect
Biotherapy	aptamer interacts with surface markers and triggers intracellular signaling of cancer cells^32^	induces activation of signaling pathway to regulate apoptosis or death of targeted cells
Cell-selective chemotherapy	as a targeting ligand, the aptamer is linked with chemotherapeutic agents, such as Dox,^33^ or functional linkers, such as polyethylene glycol^34^	increases half-life and payload capacity of chemotherapeutic agents, with enhanced anticancer effects and fewer toxic side effects
Gene therapy	as a targeting ligand, the aptamer is linked to siRNA^29^ or miRNA,^35^ and it forms aptamer-RNA chimeras	specific delivery of siRNA or miRNA into target cells to silence pathogenic oncogenes
Targeted nanomedicine	as a targeting ligand, the aptamer is linked with nanoparticles^36^, ^37^, ^38^	increases circulation half-life, payload capacity, and target drug delivery
Immunotherapy	as a targeting ligand, aptamer directly activates immune costimulatory molecules,^39^ suppresses immune checkpoint signaling,^40^ or recruits immune cells to target cells	precisely amplifies our immune system to fight malignancies

Hematologic malignancies are types of cancer that begin in the blood-forming tissue, such as the bone marrow or the cells of the immune system. Historically, hematological malignancies comprise leukemia, lymphoma, and multiple myeloma (MM), a classification that is in accord with the WHO.^41^ Herein we focus on recent advances in the applications of aptamers as therapeutic agents of hematological malignancies.

### Applications of Aptamers in Precision Medicine Therapy of Leukemia

Leukemia is a broad term for a group of cancers originating from hematopoietic stem cells; it is characterized by uncontrolled proliferation of undifferentiated white blood cells in peripheral blood and bone marrow.^42^ As the abnormal cells cannot develop further, they accumulate over time to substitute normal blood cells; this causes various symptoms correlated with dysfunction of blood cells, including anemia, bleeding, bruising, fatigue, fever, and enhanced risk of infections.^43^ Leukemias are generally classified into four main categories, depending on the disease progression and type of blood cells that are affected: acute myeloid leukemia (AML), acute lymphoblastic leukemia (ALL), chronic myeloid leukemia (CML), and chronic lymphocytic leukemia (CLL). Currently, chemotherapy is the mainstream treatment for leukemia.^1^ However, chemotherapeutic agents lack selectivity between tumors and normal tissues, which results in serious side effects such as toxicity, limited drug concentration in cancer cells, and reduced therapeutic efficacy.^44^

#### Aptamer-Mediated Therapies of AML

AML is a type of malignant disorder originating from rapid clonal expansion of undifferentiated myeloid precursors,^45^ and it is currently treated with chemotherapy, despite the possibility of causing severe non-specific toxicity to normal tissues.^46^ Recent studies demonstrated that aptamer-mediated precision therapy has considerable efficacy in the treatment of AML. AS1411, the first ssDNA aptamer to undergo clinical trials, proved to be safe and well tolerated in patients;^47^, ^48^ it can specifically target nucleolin, which is overexpressed in the cytoplasm and on membranes of various cancer cells, including AML.^49^ Chen et al.^50^ reported that AS1411 significantly inhibited cell growth and reduced viability of the AML cell line MV4-11 and AML patient cells. Additional experiments revealed the mechanism by which AS1411 interfered with bcl-2 mRNA, an important anti-apoptotic factor, playing a crucial role in the stabilization of nucleolin.^50^ The phase I clinical trial, completed in 2006, confirmed the safety and good tolerance of AS1411.^51^ Furthermore, the phase II study (ClinicalTrials.gov: NCT00512083) demonstrated the synergistic anticancer effects of AS1411 with cytarabine in the treatment of AML, thus rendering it a promising candidate for AML therapy.^47^

In addition to their role as direct, targeted biotherapeutics, aptamers can function as effective vehicles carrying drugs and target biomarkers on hematopoietic cells.^52^ Recently, Zhao et al.^53^ developed a DNA aptamer specifically targeting CD117, a biomarker highly expressed by certain AML cells.^54^, ^55^ For the therapeutic study, the aptamer was conjugated with methotrexate (MTX) to generate Apt-MTX conjugates. This Apt-MTX conjugate showed significantly enhanced inhibition of cellular growth, triggered cell apoptosis, and induced G1 phase cell-cycle arrest of the AML cell line HEL and primary AML cells from patients, compared to MTX treatment alone, with negligible toxicity on off-target cells.^53^ Another well-characterized plasma membrane biomarker, CD123, which is highly expressed on 45%–95% of AML cells,^56^ has been associated with increased resistance to chemotherapeutic drugs,^57^ higher relapse rates, or poor prognosis^58^ of AML, thus rendering it an ideal target for AML treatment. Wu et al.^59^ developed the first CD123 aptamers (dubbed ZW25 and CY30), and they designed a novel CD123-aptamer-mediated targeted drug train (TDT) with effective, biocompatible, and high drug-loading capacity. These two CD123 aptamers bind to an epitope of CD123 peptide and CD123(+) AML cells with high specificity and minimal cross-reactivity to other proteins, such as albumin, IgG, or trypsin.^59^ The TDT transported a high load of Doxorubicin (Dox) to CD123+ cells with substantial efficacy, while significantly reducing drug toxicity to CD123− cells *in vitro*.^58^ In addition, the TDT inhibited tumor growth of a mouse xenograft tumor model *in vivo* and prolonged their survival.^59^ In summary, these results suggest that aptamer and aptamer-mediated chemotherapies have high potential to selectively deliver cytotoxic agents to target cells, opening a new avenue in the precision treatment of AML.

#### Aptamer-Mediated Therapies of ALL

ALL is an aggressive neoplasm stemming from uncontrolled proliferation of immature T or B lymphoblasts in bone marrow.^60^ Conventional chemotherapeutic treatments for ALL have shown limited efficacy. However, to date, non-specific toxicity toward normal tissues and relapses in one-fifth of the cases still remain big challenges for ALL patients.^61^ In recent years, the applications of aptamer-mediated targeted therapies have increased exponentially.

An important milestone in the field was the development of Sgc8c-7, an ssDNA aptamer that Shangguan et al.^62^ developed in 2006. Aptamer Sgc8c-7 specifically targets protein tyrosine kinase 7, which is highly expressed on the membrane of T-ALL cell line CCRF-CEM,^62^ thus providing excellent possibilities for more effective and precise treatment of ALL. Pioneering work by Huang et al.^33^ showed that conjugation of Dox to aptamer Sgc8c-7 resulted in highly efficient targeted delivery of Dox to CCRF-CEM cells, with minimum uptake by off-target cells; the aforementioned results show the advantages of aptamers in clinical applications. Besides linking to chemotherapeutic drugs, aptamer conjugates with new anticancer agents have also been extensively used in cancer treatment. Recently, photosensitizers emerged as a new group of anticancer agents because they can be activated by light irradiation to generate reactive oxygen species.^63^, ^64^ However, photosensitizers showed insufficient localization at the target sites *in vivo* due to a lack of cellular specificity. Wang et al.^65^ successfully overcame this limitation by linking aptamer Sgc8c-7 with photosensitizer Ce6; the conjugate aptamer significantly increased selective binding and death of CCRF-CEM cells.

In addition, nowadays, the use of nanoparticles, a promising approach in targeted medicine, is gradually gaining momentum in the treatment of ALL. Nanoparticles have good biocompatibility, large surfaces for enhanced aptamer loading, and uniform size and shape for excellent biodistribution. These characteristics prolong nanoparticle half-life *in vivo* and increase payload capacity of linked agents.^18^ N-Heterocyclic carbenes (NHCs) are a class of organic compounds that can stabilize metals in air, heat, water, and acid through strong bonding.^66^ In recent years, NHC conjugates with gold nanoparticles (NHC-Au) have attracted our attention as a new group of potential anticancer agents. NHC-Au complexes are physically stable, and they exhibit remarkable cytotoxicity because they can efficiently inhibit growth and induce apoptosis of cancer cells.^67^, ^68^ However, a common disadvantage of metal-based drugs entails their non-specific interactions with normal cells or tissues.^69^ Improvement of the cellular selectivity of NHC-Au complexes prevented these undesired interactions. Recently, Niu et al.^70^ reported that covalent conjugates of aptamer Sgc8c-7 to NHC-Au complexes selectively bound to CCRF-CEM cell lines and were specifically internalized into cells, without interacting with off-target cells. Additionally, a significantly higher cytotoxicity was observed against CCRF-CEM cells when they were treated with Sgc8c-7 conjugated to NHC-Au as compared to treatment with NHC-Au complexes alone; this result indicates that Sgc8c-7 can mediate specific and efficient delivery of NHC-Au to target cells, thus killing cancer cells with high efficiency.^70^ In addition, Luo et al.^71^ developed a smart drug carrier by assembling Sgc8c aptamer, Dox, and hairpin DNA complexes on the surface of gold nanoparticles. The aptamer-functionalized nanoconjugates specifically bound to CCRF-CEM cells, released encapsulated Dox with hairpin DNA when exposed to laser illumination, and specifically killed cancer cells.^71^

In conclusion, aptamer Sgc8c-7 functions as an important vehicle for targeted medicine therapy of ALL via conjugation to chemotherapeutic drugs, photosensitizers, and nanoparticles, thereby enhancing their selective cytotoxicity without damaging normal tissues. Furthermore, it achieves highly specific synergistic anticancer effects when combined with other anticancer agents, which underscores the high potential of aptamers in future clinical applications to treat ALL.

#### Aptamer-Mediated Therapies of CML

CML is a slowly progressing myeloproliferative neoplasm that originates from abnormal pluripotent bone marrow stem cells, and it is consistently associated with BCR-ABL fusion gene located in the Philadelphia (Ph) chromosome.^72^ Currently, precision therapy blocking the activity of ABL tyrosine kinase with the tyrosine kinase inhibitor (TKI) Imatinib is the first-line treatment for newly diagnosed CML patients.^73^, ^74^ However, in nearly 40% of CML patients, Imatinib exhibits inadequate efficacy or loss of previously obtained response;^75^ thus, new alternatives for treatment are necessary. Ping et al.^76^ recently developed an aptamer that can specifically target BCR-ABL fusion gene, and they linked it with small interfering RNA (siRNA) to induce gene silencing. The studies showed that this aptamer-siRNA nucleic acid chimera significantly inhibited cellular growth and induced apoptosis of CML cell line K562 only,^76^ indicating its potential therapeutic value in CML treatment.

Besides impacting typical genes, aptamers exhibit anticancer activity by interfering with signaling pathways. Beta-arrestins, a family of ubiquitous cellular scaffold proteins, are important signaling adaptors, facilitating activation of various pathways^77^, ^78^ that are closely correlated to cellular proliferation, differentiation, or apoptosis^79^ and several tumorigenic events.^80^, ^81^ It was demonstrated that β-arrestin 2, a member of the β-arrestin family, was critical in the initiation and progression of CML via modulation of the Hh/Smo and Wnt/Fz pathways; this indicates that β-arrestin 2 is a viable target in treating CML.^82^ Based on these findings, Kotula et al.^83^ screened an aptamer that can specifically bind to β-arrestin 2. The aptamer specifically disrupted β-arrestin 2-signaling complexes, and thus it inhibited the downstream signaling processes of both Hh/Smo and Wnt/Fz pathways; these effects finally led to impaired tumorigenic growth of CML cell line K562 and patient-derived samples.^83^ Aptamers targeting specific genes and signaling molecules, involved in the development of CML, showed considerable effects in precision medicine therapy of the disease. Given that use of TKIs presently remains the conventional treatment for CML, the combination of aptamers with TKIs is an important route to achieve enhanced therapeutic effects in the future.

#### Aptamer-Mediated Therapies of CLL

CLL is a neoplasm composed of immune-incompetent and clones of self-renewing B cells,^84^ which relentlessly accumulate in the peripheral blood, bone marrow, spleen, and lymph nodes.^85^ It accounts for 25% of all leukemias, and it ranks as the most common lymphoid malignancy in western countries, with a highly variable clinical course and prognosis.^86^ Conventional chemotherapies only eliminate CLL cells in peripheral blood, whereas those in bone marrow remain unaffected, thus leaving CLL incurable. An important proliferation mechanism involves bone marrow stromal cells, which constitutively secrete a type of chemokine named CXCL12; it recruits immune cells via interaction with its receptor CXCR4, which is highly expressed on the surface of CLL cells.^87^ CXCL12 and CXCR4 signaling has been demonstrated to promote interaction between CLL cells and the bone marrow microenvironment, and it enhances homing and retention of CLL cells in bone marrow.^87^ Thus, the inhibition of such tumor-supporting pathways could potentially enhance the therapeutic effects of chemotherapy on CLL and totally eradicate CLL cells in bone marrow.

Taking into consideration the previous findings, the RNA aptamer NOX-A12 was developed; it comprises 45 ribonucleotides and acts as the specific antagonist of CXCL12.^88^ NOX-A12 disrupted binding of CXCL12 to CXCR4, and it mobilized CLL cells from the protective bone marrow microenvironments to peripheral blood, thus sensitizing CLL cells to chemotherapeutic attack and triggering cellular apoptosis.^89^ The phase IIa clinical study of NOX-A12 in combination with Bendamustine and Rituximab in the treatment of relapsed CLL (ClinicalTrials.gov: NCT01486797) showed that the administration of NOX-A12 was superior to chemotherapy with respect to overall response rate and complete remission of CLL; these results demonstrate the promising perspective of aptamers in the treatment of chemotherapy-resistant CLL.^90^ In summary, aptamers targeting the CLL microenvironment can effectively enhance sensitivity and cytotoxicity of chemotherapeutic drugs on CLL cells. The combination of chemotherapeutic drugs with aptamers is a very promising route in the future treatment of CLL.

### Applications of Aptamers in Precision Medicine Therapy of Lymphoma

Lymphomas comprise a group of neoplasms that develop from the lymphatic system, and they are characterized by uncontrolled proliferation of lymphocytes.^91^ Typical signs and symptoms of lymphoma include enlarged painless lymph nodes, fever, drenching sweats, unintended weight loss, and constant fatigue.^92^ Based on the cell histology, genetic mutations, expression of certain genes, and other characteristics, lymphomas can be generally classified into Hodgkin’s lymphoma and non-Hodgkin’s lymphoma (NHL) with T or B cell subtypes.^93^, ^94^, ^95^ In this review, we discuss the progress of published reports on aptamer-mediated precision medicine therapy of lymphoma.

#### Aptamer-Mediated Therapy of T Cell Lymphomas

T cell lymphomas affect T cells at different stages, and they account for about one in ten cases of NHL.^43^ Anaplastic large cell lymphoma (ALCL) is an aggressive T cell lymphoma characterized by cohesive proliferation of large pleomorphic lymphoma cells, abnormal activation of anaplastic lymphoma kinase (ALK) oncogene, and high-level expression of CD30 molecules.^96^ CD30 is a transmembrane protein receptor of the tumor necrosis factor (TNF) family, and it is normally expressed in activated T cells.^97^, ^98^ CD30 protein targeting appears to be an attractive and rational approach for the diagnosis and treatment of ALCL.

In 2004, Mori et al.^99^ first developed an RNA aptamer that can specifically target CD30. Recently, *in vivo* RNA delivery tools, such as polyethyleneimine (PEI), showed high cellular transfection efficiency and high buffering capacity.^100^, ^101^, ^102^ Zhao et al.^103^ designed a PEI-citrate nanocomplex, which was equipped with CD30 aptamer and loaded with siRNA of ALK oncogene. In general, chromosomal translocation of the ALK gene was observed in 50%–85% of ALCL patients,^104^ and the knockout of ALK gene significantly increased the death rate of ALCL cells.^105^, ^106^ The *in vitro* studies revealed that the nanocomplex selectively bound to CD30(+) ALCL cells, effectively silenced cell ALK oncogene, and triggered growth arrest as well as apoptosis of ALCL cells Karpas 299.^103^ Also, Zhao et al.^107^ developed another nanoscale drug delivery system, comprising a hollow gold nanosphere equipped with RNA CD30 aptamer on the surface and Dox inside the nanosphere. This gold nanosphere was stable under normal biological conditions (pH 7.4), but it became ultrasensitive at low pH and rapidly released 80% of loaded Dox at pH 5.0. Upon aptamer-mediated biomarker interaction, the gold nanospheres were specifically internalized in ALCL cells and trafficked into lysosomes, where the low pH triggered effective release of the loaded Dox; thus, the aptamer nanospheres selectively killed ALCL cells without damaging off-target cells.^107^

In contrast to DNA oligonucleotides, RNA aptamers are unstable because they can be rapidly digested by serum nucleases, a fact that largely limits their applications under physiological conditions.^108^ Parekh et al.^109^ developed a highly stable ssDNA aptamer targeting CD30 biomarkers on lymphoma cell lines (Karpas 299, SU-DHL-1, HDLM2, and KMH2), with nanomolar affinity and specificity. This CD30 aptamer is stable in serum and functions well in complex biological fluids. Its multimer form exhibits specific promotion and induction of a cell-signaling pathway related to the apoptosis of target cells *in vitro*.^109^ Furthermore, Zhao et al.^110^ developed a multifunctional aptamer-nanomedicine (Apt-NMed) simultaneously achieving targeted chemotherapy and gene therapy of ALCL. Apt-NMed was formulated by the self-assembly of ssDNA aptamer and ALK-specific siRNA followed by self-loading of the chemotherapeutic drug Dox.^110^ Intracellular delivery of Apt-NMed triggered rapid Dox release and intracellular ALK-specific siRNA delivery, resulting in the combined chemotherapeutic and oncogene-silencing effects.^110^ Animal model studies demonstrated specific accumulation of Apt-NMed in ALCL tumor sites, induction of higher inhibition of ALCL tumor growth, and improved survival of the treated mice; Apt-NMed did not affect off-target tumors in the same xenograft mouse, thus opening a new avenue for precision therapy of ALCL.^110^

In addition, we investigated unique aptamer-equipped natural killer (NK) cells for immunotherapy of ALCL (S.Y., unpublished data). The study showed that the aptamer-equipped NK cells specifically recognized and captured CD30(+) ACLC cells by increasing NK and target cell clusters; this resulted in increased killing efficiency of target cells. To sum up, the CD30-specific aptamer functions as an excellent guiding tool to efficiently deliver anticancer agents or immune cells to target cells or to specifically trigger an intracellular signaling pathway by binding to its receptors on lymphoma cells. These results lead to a wide range of successful precision medicines to treat T cell lymphoma.

#### Aptamer-Mediated Therapies of B Cell Lymphomas

B cell lymphomas comprise a group of malignancies resulting from the clonal proliferation of mature B lymphocytes.^111^ They include most NHLs and are more prevalent than T cell lymphomas.^112^ In general, B cell lymphomas express specific surface biomarkers that are restricted to a single light-chain type and a specific variable region. These surface markers are considered tumor specific and distinguish tumors from normal cells.^113^ Recently, studies on precision therapy studies of B cell lymphomas based on cell-specific biomarkers were conducted.^114^, ^115^, ^116^ It is well known that B cell lymphomas express only one type of surface Ig light chain (kappa or lambda); these chains are considered the gold standard biomarkers for diagnosis and potential targets for precision therapy of lymphoma.^117^, ^118^ Li et al.^119^ developed a ssDNA aptamer that can specifically target Maver-1 lymphoma (B cell lymphoma) cells and Ig lambda-like polypeptide. The functional studies revealed selective internalization of aptamers into Maver-1 lymphoma cells, triggering cell cycle S-phase arrest and cellular growth inhibition. Importantly, the aptamer-induced S-phase arrest and chemotherapeutic treatment with cytarabine had a notable synergistic effect on killing Maver-1 cells. These results demonstrate the importance of aptamers in synergistic biotherapy via cell cycle regulation and chemotherapy of lymphoma.^119^

CD20, a B cell-differentiating antigen, is selectively expressed on the surface of mature and malignant B cells.^120^ Currently, the FDA has approved several anti-CD20 monoclonal antibodies (mAbs), such as Rituximab (RTX, Rituxan), Ofatumumab (Arzerra), Obinutuzumab (Gazyva), the radio-conjugates Bexxar (Tositumomab-I131), and Zevalin (Ibritumomab tiuxetan).^121^, ^122^ Although clinical treatments with mAbs have been satisfactory, serious side effects, such as allergic reactions^123^ and cross-reaction with normal B cells,^124^ remain considerable challenges. Aptamers show much lower immunogenicity and are considerably more tolerable *in vivo*.^107^ Recently, Wu et al.^125^ successfully screened a DNA aptamer with significantly higher binding affinity for CD20, as compared to mAb RTX. Besides, RTX induced caspase-dependent apoptosis of NHL cells via crosslinking of cellular CD20; this indicates its potential role in the immunotherapy of B cell lymphomas.^125^

B cell-activating factor receptor (BAFF-R) is another important tumor-specific marker in B cell lymphoma. Essentially, it enhances maturation, survival proliferation, and maintenance of peripheral B cells by interacting with its ligand B cell-activating factor (BAFF).^126^, ^127^ Overexpression of BAFF-R is found in several types of B cell lymphomas,^128^ including the most common type of diffuse large B cell lymphoma (DLBCL)^129^ and a rare, aggressive type of mantle cell lymphoma.^130^ Previous studies demonstrated that BAFF-R can promote the proliferation of malignant B cells.^131^ On the basis of this finding, Zhou et al.^132^ successfully developed a 2′-F-modified RNA aptamer that can specifically target BAFF-R on malignant B cells with high affinity. This RNA aptamer efficiently blocked B cell proliferation signals, mediated by BAFF/BAFF-R, by competing with BAFF for its receptor.^132^ Moreover, it functions as a specific vehicle to deliver siRNA for gene-silencing purposes, indicating the potential role of aptamers in targeted therapy of B cell malignancies.^132^

Dysregulation of the c-Myc gene and rearrangement of Ig heavy chains are important mechanisms, leading to the progression of certain types of B cell malignancies, such as Burkitt’s lymphoma.^133^ Tang et al.^134^ developed a DNA aptamer (TD05), which binds to the Ig heavy chain of Burkitt’s lymphoma cell line Ramos with high affinity and specificity. As previously reported, photosensitizers are ideal cytotoxic agents for cancer treatment. Mallikaratchy et al.^135^ first linked TD05 to a photosensitizer, and they performed selective photodynamic therapy of targeted cancers. The TD05 aptamer-photosensitizer chimera successfully targeted Ramos cells *in vitro*, and it effectively generated more free radicals, triggering cellular death upon illumination.^135^ In addition, Wu et al.^136^ modified TD05 by attaching a lipid tail to its end, and they formed a self-assembled aptamer-micelle nanostructure. This unique nanostructure had several advantages compared to the aptamer alone, including faster recognition by targets, lower release rate after binding to the targets, and considerable dynamic binding affinity to target cells in a flow channel system mimicking drug delivery in the bloodstream. These findings show the advantages of aptamer-micelle nanostructures in drug delivery to B cell lymphomas *in vivo*.^136^

Besides targeting surface-specific markers, aptamers can attack B cell lymphoma by modulating the immune system. In recent decades, substantial efforts have been made to promote the use of T cells in cancer immunotherapy by modulating immune checkpoints or costimulatory molecules. CD28 is the main costimulatory receptor constitutively expressed on T cells, and it interacts with its ligands B7-1 and B7-2 expressed on antigen-presenting cells (APCs), such as dendritic cells, macrophages, and B cells.^137^ Recently, it was demonstrated that the B7-1- or B7-2-CD28 pathway on APCs is the strongest costimulatory signal for the full activation of T cells.^138^ Pastor et al.^138^ developed an aptamer that activated CD28; the investigators demonstrated that it boosted T cell immune response against a murine B cell lymphoma model and considerably prolonged the overall survival of mice.

In addition, aptamers can interfere with lymphoma growth by disrupting interactions of functional cellular molecules. Nucleolin, which is overexpressed in the cytoplasm of various cancer cells, including DLBCL,^49^ interacts with the DNA repair enzyme complex topoisomerase-II-alpha (TopIIA). It was reported that the interaction between nucleolin and TopIIA was essential in blocking DNA damage and cellular apoptosis and abrogation of nucleolin-sensitized DLBCL cells to TopIIA-targeting agents.^139^ Targeting nucleolin with the specific aptamer AS1411 significantly increased the cytotoxicity of Dox toward DLBCL cells; this finding shows the clinical importance of R-CHOP-based therapy in improving the prognosis of DLBCL patients.^139^

In summary, aptamer-mediated precision therapy of B cell lymphoma mainly interferes with cell-specific biomarkers or cellular molecules, and it also modulates the immune system, resulting in direct, remarkable cancer suppression effects.

### Applications of Aptamers in Precision Medicine Therapy of MM

MM is a plasma cell neoplasm characterized by clonal proliferation of malignant plasma cells in the bone marrow, monoclonal Ig accumulation in blood or urine, and associated organ dysfunction.^140^ Despite conventional therapeutics such as Bortezomib,^141^ MM still remains an incurable disease with poor prognosis for the majority of patients, and ongoing research is required to find additional therapeutic approaches.^142^

In recent years, aptamer-based precision medicine has shown promising results in the treatment of MM. CD38, a type II transmembrane glycoprotein that is widely expressed on plasma cells, is considered an important biomarker for diagnosis and a promising target for precision therapy of MM.^143^ Wen et al.^144^ developed a CD38-specific ssDNA aptamer, capable of targeting MM cells with high affinity. The authors conjugated this aptamer with Dox to form Apt-Dox conjugates (ApDCs); they observed specific internalization of ApDCs in MM cells and continuous trafficking into lysosomes, where the low pH microenvironment triggered structural changes, leading to the rapid release of Dox. The released Dox (from ApDCs) specifically killed MM cells without affecting off-target cells.^144^ The therapeutic potential of ApDCs was also demonstrated by tumor growth inhibition and the lack of toxicity in xenograft mice.^144^ Furthermore, a ssDNA aptamer specifically binding to Annexin A2 (ANXA2), the protein closely related to proliferation and adhesion of MM, has been developed.^145^ The binding specificity of the aptamer was confirmed *in vivo* in nude mice xenografts with MM cells and MM bone marrow aspirates. It was demonstrated that the aptamer blocked MM cell proliferation induced by ANXA2, thereby providing a promising candidate for MM diagnosis and treatment.^145^ In summary, aptamer-mediated therapeutic approaches, via targeting cell proliferation pathways or specific surface markers, have shown promising effects in the precision treatment of MM.

### Conclusions

The newly developed aptamer technology has shown promising results in precision medicine therapy of hematological malignancies (summarized in Table 2). Currently, patients diagnosed with hematological malignancies are primarily treated by standard chemotherapy.^1^ However, the clinical outcomes of chemotherapeutic treatments showed only minimal improvement over the past three decades due to unavoidable side effects induced by chemotherapeutic agents; these include toxicity to normal tissues, limited drug concentration, and high relapse rates.^44^

**Table 2:** Aptamer-Mediated Precision Medicine Therapy Applications in Hematological Malignancies

Disease	Biomarker	Development	Therapy	Application
AML	nucleolin	by nonantisense synthesize based on observations that guanosine-rich oligonucleotides possessed antiproliferative properties against cancer cells	biotherapy	synergetic anticancer effect of AS1411 with cytarabine in treatment of AML during phase II study (NCT00512083)^47^
	CD117	SELEX from whole cell	cell-selective chemotherapy	cellular growth inhibition, apoptosis, and cell-cycle arrest of AML cell line, and patient sample by Apt-MTX conjugates^53^
	CD123	SELEX from peptide	cell-selective chemotherapy	cellular cytotoxicity of AML cell line, tumor growth inhibition and prolonged survival of AML xenograft mouse model via Apt-Dox conjugates^59^
ALL	PTK7	SELEX from whole cell	cell-selective chemotherapy	specific enhanced cellular cytotoxicity of ALL cell line via Apt-Dox conjugates^33^ and Apt-photosensitizer Ce6 conjugates^65^
			nanomedicine	specific enhanced killing effects on ALL cell line via Apt-NHC-Au conjugates^70^ and Apt-Dox-hairpin DNA complexes^71^
CML	BCR-ABL	SELEX from purified protein	gene therapy	significant cellular growth inhibition and apoptosis of CML cell line induced by Apt-siRNA chimera^76^
	β-arrestins	SELEX from purified protein	biotherapy	impaired growth of CML cell line and patient-derived samples via β-arrestin 2 aptamer^83^
CLL	CXCL12	SELEX from purified protein	biotherapy	sensitizing CLL cells to chemotherapy with enhanced apoptosis rates via aptamer targeting;^89^ increased complete remission rate of aptamer-treated relapsed CLL patients; aptamers used in combination with Bendamustine and Rituximab in phase IIa clinical study (NCT01486797)^90^
T cell lymphoma	CD30	SELEX from whole cell and purified protein	gene therapy	cellular growth arrest and apoptosis triggered by Apt-siRNA-PEI citrate carrier toward target cell line^103^
			nanomedicine and cell-selective chemotherapy	significant enhancement of target cell line killing achieved by Apt-Au-Dox conjugates^107^
			cell-selective chemotherapy and gene therapies	chemotherapy and oncogene silencing of target cell lines, tumor growth inhibition, and higher survival rate of xenograft mouse models achieved by Apt-siRNA-Dox conjugates^110^
			immunotherapy	increased capturing and killing efficiency of target cell lines induced by aptamer-equipped NK cells (S.Y., unpublished data)
B cell lymphoma	Ig light chain	SELEX from whole cell and purified protein	biotherapy	cell-cycle arrest, growth inhibition, and synergistic killing effect of target cell lines by chemotherapeutics via aptamer targeting^119^
	CD20	SELEX from whole cell	biotherapy	apoptosis of target cell lines^125^
	BAFF-R	SELEX from purified protein	biotherapy and gene therapy	inhibition of cellular proliferation of target cell lines by direct aptamer binding and oncogene silencing by Apt-siRNA conjugates^132^
	Ig heavy chain	SELEX from whole cell	cell-selective chemotherapy	induction of target cell line death upon illumination of the Apt-photosensitizer chimera^135^
	CD28	SELEX from purified protein	immunotherapy	enhanced cellular immune response against lymphoma and prolonged survival of mice^138^
	nucleolin	by nonantisense synthesize based on observations that guanosine-rich oligonucleotides possessed antiproliferative properties against cancer cells	biotherapy	facilitated chemotherapy against target cell lines via aptamers^139^
Multiple myeloma	CD38	SELEX from whole cell and purified protein	cell-selective chemotherapy	increased cellular death rates of target cell lines via Apt-Dox conjugate targeting^144^
	Annexin A2	SELEX from purified protein	biotherapy	blocked proliferation of MM cells induced by Annexin A2 nude mouse and bone marrow aspirates^145^

Aptamer-mediated precision therapy showed superior outcomes in clinical applications as compared to chemotherapy. First, aptamer-mediated therapies cover a vast spectrum of targets, including extracellular tumor-specific markers, intracellular oncogenes, dysregulated proteins, or signaling pathway-related factors, thus providing more options to treat and cure cancers. Given the complex pathogenesis of leukemias, lymphomas, and MM, the development of treatments based on widespread targeting is very valuable. Second, aptamers can be easily modified by the conjugation of different anticancer agents, such as chemotherapeutic drugs, nanoparticles, siRNAs, and guiding immune cells. The flexibility of modifying aptamers coordinates well with their wide spectrum of targets, and it leads to enhanced anticancer effects as compared to chemotherapeutic drugs. Finally, aptamers are small oligonucleotides with high affinity and specificity for their targets and fewer side effects to normal tissues. The high safety and tolerance of aptamer-mediated treatments are of great importance, especially in the treatments of leukemia where cancer cells are diffusely distributed in the tissues.

Aptamer-mediated treatments provide an advantageous approach compared to conventional therapies, and they present a promising perspective in clinical applications given their aforementioned unique properties and development potential. However, several challenges remain to be addressed. Unmodified RNA aptamers are vulnerable to nuclease-mediated degradation in the bloodstream, and thus methods to improve the *in vivo* biostability of aptamers are urgently needed. Despite chemical modifications of RNA aptamers, including changes to the phosphodiester backbone or PEGylation to improve their stability *in vivo*,^146^, ^147^ their effectiveness has remained limited. Moreover, aptamer-drug conjugates may display different pharmacokinetics, biodistribution, and tolerability *in vivo*. Therefore, maintaining the same efficiency of aptamer-drug conjugates from bench to bedside remains a considerable challenge. Also, aptamer-based products initially had limited commercial success in the markets because aptamer technology was new, and relevant systematic academic studies on pharmacokinetics and pharmacodynamics were not available.^18^ The aforementioned issues should be addressed prior to the future application of aptamers as dominant therapeutics in the clinic.

In summary, aptamers have opened an attractive and promising venue of precision medicine in cancer treatment. Next-generation aptamer-based therapeutics with superior biological functions will emerge as new aptamers are developed. Additional aptamer-based therapeutic approaches, such as treatment of leukemia or lymphoma in combination with stem cell transplantation or CAR-T cell adoptive transfer, may be explored and applied in the future. As new clinical improvements are achieved, the number of successful aptamer-based applications in cancer therapy will certainly increase, thereby providing a strong incentive to further develop this promising class of cancer therapeutics.

## Author Contributions

S.Y. wrote the manuscript. Y.Z., H.L., L.X., and Z.D. revised the manuscript. W.H., Y.L., and W.J. drew the figures and summarized the tables.

## Conflicts of Interest

The authors have no conflicts of interest to disclose.
